# Effects of the adiponectin mimetic compound ALY688 on glucose and fat metabolism in visceral and subcutaneous rat adipocytes

**DOI:** 10.1080/21623945.2020.1817230

**Published:** 2020-09-08

**Authors:** Daniel Da Eira, Shailee Jani, Hyekyoung Sung, Gary Sweeney, Rolando B. Ceddia

**Affiliations:** a School of Kinesiology and Health Science, York University, North York, Canada; b Department of Biology, York University, North York, Canada

**Keywords:** Adipokines, AMPK, adipocyte metabolism, glucose uptake, lipogenesis, lipolysis, insulin, AdipoR1, AdipoR2

## Abstract

Adiponectin regulates white adipose tissue (WAT) metabolism and promotes insulin-sensitizing and anti-atherosclerotic effects *in vivo*. In this context, small molecule adiponectin receptor agonists have become of great therapeutic value for the treatment of metabolic diseases. Here, we investigated the effects of the adiponectin mimetic compound ALY688 on WAT metabolism. To accomplish this, rat epididymal (Epid) and subcutaneous inguinal (Sc Ing) adipocytes were isolated and incubated with ALY688. Subsequently, several parameters of glucose and fat metabolism were assessed. ALY688 promoted AMP-activated protein kinase (AMPK) and acetyl-CoA carboxylase (ACC) phosphorylation, enhanced glucose oxidation, and suppressed fat oxidation in adipocytes from both fat depots. ALY688 did not affect basal and insulin-stimulated rates of glucose uptake, glucose incorporation into lipids, and AKT_Ser473_ and p38 mitogen-activated protein kinase (MAPK) phosphorylations in either Epid or Sc Ing adipocytes. ALY688 did not alter basal lipolysis in Epid and Sc Ing adipocytes, but it enhanced isoproterenol-induced lipolysis in Epid adipocytes. Adiponectin receptor 2 (AdipoR2) mRNA was the prevalent isoform expressed in all adipocytes, and Epid adipocytes displayed significantly higher AdipoR2 mRNA expression than Sc Ing adipocytes. In conclusion, ALY688 can regulate adiposity and affect glycaemic control by altering substrate portioning in the WAT in a fat depot-specific manner.

## Introduction

Maintenance of whole-body glucose homoeostasis depends on the coordinated action of multiple hormones that allow the organism to respond to daily oscillations in the availability of dietary carbohydrate and other macronutrients [1]. In this context, the adipose tissue plays an important role in maintaining whole-body glucose homoeostasis. It does so by serving as a compartment where energy can be stored or mobilized and also by secreting hormones that regulate glucose and fat metabolism in multiple organs and tissues [2]. To date, many bioactive adipose-derived factors (adipokines) have been identified [2]. Among them is adiponectin, a secreted protein abundantly present in plasma, that has been demonstrated to exert potent insulin sensitizing, glucose lowering, and lipid catabolizing functions in peripheral tissues [3]. Two adiponectin receptors (AdipoRs) have been cloned (AdipoR1 and AdipoR2), which are expressed in various tissues including liver, skeletal muscle, white adipose tissue (WAT), hypothalamus, and vasculature [4]. The metabolic effects of adiponectin vary in a tissue-specific manner, depending in part on tissue-specific expression of AdipoR1 and AdipoR2 as well as on cell-specific, intracellular signalling pathways [4]. For instance, AdipoR1 is abundant in skeletal muscles and macrophages, whereas WAT and the vasculature are rich in AdipoR2. The liver abundantly expresses both AdipoRs [4].

**Figure 1.**
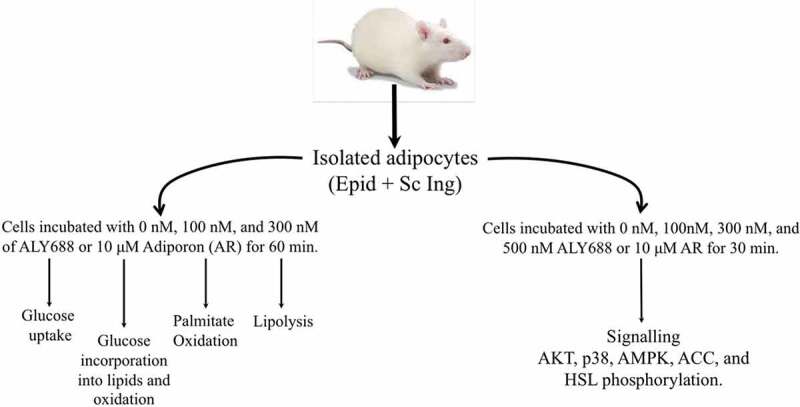
Schematic representation of the experimental design

Once bound to its receptors, adiponectin has been shown to promote the activation of AMP-activated protein kinase (AMPK) and peroxisome proliferator-activated receptor α (PPARα) [4,5]. In liver and skeletal muscles, activated AMPK phosphorylates acetyl-CoA carboxylase (ACC) and leads to enhanced mitochondrial import and oxidation of long-chain fatty acids [6,7]. Adiponectin has also been shown to exert anti-lipotoxic effects through the induction of the transcriptional effects of PPARα[8], culminating in the downregulation of lipid synthesis enzymes, the elevation in carnitine palmitoyltransferase 1 (CPT1) levels, and the enhanced production and secretion of HDL by the liver [9]. In the WAT, adiponectin has been demonstrated to elevate the fat-storing capacity of adipocytes by promoting glucose uptake [10] and lipogenesis [11] and by decreasing lipolysis [12], although the enhancement of fat oxidation has also been cited as an effect of adiponectin in fat cells [3]. The opposing lipogenic and fatty acid oxidative effects of adiponectin on adipocytes have been proposed to confer metabolic flexibility to the WAT, which contributes to minimizing ectopic lipid deposition in non-adipose tissues, preventing adipose inflammation and fibrosis, and maintaining healthy WAT expansion [3,13]. However, the findings that support an elevation in fatty acid oxidation induced by adiponectin originated from studies conducted in C_2_C_12_ muscle cells [7,8,14], isolated mouse skeletal muscles [6,8,14,15], mouse hepatocytes [6], and hepa-1-6 hepatocytes in culture [14]. To the best of our knowledge, no direct evidence exists that adiponectin indeed promotes fatty acid oxidation in adipocytes. Disruption of both AdipoR1 and AdipoR2 has been reported to significantly decrease the expression of genes encoding for *Cat* and *Sod* and increase oxidative stress in WAT, but there were no reports of alterations in the capacity of the WAT to oxidize fat in mice lacking AdipoR1 and AdipoR2 [16]. Thus, it is likely that the metabolic responses of WAT to adiponectin differ from those of skeletal muscles and liver with respect to fatty acid oxidation.

Reduced circulating levels of adiponectin and impaired AdipoR action are found in obesity, insulin resistance, type 2 diabetes, and atherosclerosis [5]. Therefore, increasing adiponectin production and/or adiponectin signalling are attractive targets for therapeutic interventions for the prevention or treatment of obesity-related derangements in metabolism. In fact, a small-molecule, AdipoR-activating compound named AdipoRon (AR), has been reported to improve insulin resistance and prolong lifespan in mice consuming a high-fat diet [17]. These findings in rodents have provided support for the development of other adiponectin mimetic compounds to target substrate metabolism in organs that play major roles in regulating whole-body glucose and lipid homoeostasis in humans. In this context, a peptide-based adiponectin mimetic named ALY688 (also referred to as ADP355) has been developed [18]. The compound has been shown to bind to both AdipoR1 and AdipoR2 and induce adiponectin-specific signalling in several cell types [19]. A potential advantage of peptide mimetics is that they can provide great potency and specificity compared with small molecule mimetics.

Because the WAT plays an important role in the regulation of glucose and lipid homoeostasis[1] and only a few studies have focused on the effects of adiponectin on this tissue, we assessed the effects of ALY688 as an adiponectin mimetic on glucose uptake and fatty acid oxidation in isolated white adipocytes. Also, due to metabolic differences between visceral (Vc) and subcutaneous (Sc) fat depots and their implications to obesity-related metabolic diseases [20], adipocytes isolated from epididymal (Epid) and Sc inguinal (Sc Ing) fat depots were used. We assessed the potential direct effects of ALY688 on glucose uptake, incorporation of glucose into lipids, fatty acid oxidation, and lipolysis, which are major pathways by which adipocytes handle glucose and fatty acids. We also measured the phosphorylation of AMPK, ACC, AKT, and p38 mitogen-activated protein kinase (p38MAPK), which are signalling pathways by which this adiponectin mimetic compound could potentially regulate metabolism in the WAT. Here, we provide evidence that despite enhancing AMPK phosphorylation in Epid and Sc Ing adipocytes, ALY688 reduced fat oxidation and did not affect basal or insulin-stimulated glucose uptake. However, it shifted glucose metabolism towards oxidation and enhanced adrenergic-stimulated lipolysis in a depot-specific manner.

## Material and methods

### Reagents

Type II collagenase, isoproterenol, fatty acid (FA)-free bovine serum albumin (BSA), palmitic acid, and the free glycerol determination kit were purchased from Sigma (St. Louis, MO, USA). [1-^14^C] palmitic acid and D-[U-^14^C] glucose were purchased from American Radiolabeled Chemicals (St. Louis, MO, USA). Protease (cOmplete Ultra Tablets) and phosphatase (PhosSTOP) inhibitors were obtained from Roche Diagnostics GmbH (Mannheim, Germany). The β-actin (Cat # 4067), AMPK (Cat # 2532), pAMPK_Thr172_ (Cat # 2535), AKT (Cat # 9272), pAKT_Ser473_ (Cat # 9271), p38 (Cat # 9212), pp38 (Cat # 9211), hormone-sensitive lipase (HSL; Cat # 4107), and pHSL_Ser660_ (Cat # 4126) antibodies were purchased from Cell Signalling (Danvers, MA, USA). The ACC (Cat # ab45174) and pACC_Ser79_ (Cat # ab68191) antibodies were purchased from Abcam (Toronto, ON, Canada). ALY688 was provided by Allysta Pharmaceuticals, Belmont, CA, USA.

### Animals

Adipose tissue from the subcutaneous inguinal (Sc Ing) and epidydimal (Epid) fat pads were extracted from male albino rats (Wistar strain weighing ~250 g, 50–60 days old) under anaesthesia, and subsequently processed for cell isolation (Figure 1). All experiments were approved by the Animal Care Committee at York University. (York University Animal Care Committee, YUACC, permit number 2016–5) and performed strictly in accordance with the YUACC guidelines. All surgery was performed under ketamine/xylazine anaesthesia, and all efforts were made to minimize suffering.

### Adipocyte isolation

Adipocyte isolation from the Epid and Sc Ing fat pads (Figure 1) was performed as previously described [21]. Briefly, the adipose tissue was minced in Krebs-Ringer bicarbonate HEPES buffer (KRBH), containing type II collagenase (0.5 mg/ml), prepared fresh on the day of each experiment from stock solutions of salts and buffers (stored at 4°C) to give the following final concentrations: 120 mM NaCl, 4.8 mM KCl, 2.5 mM CaCl_2_, 1.2 mM KH_2_PO_4_, 1.2 mM MgSO_4_, 15 mM NaHCO_3_, and 30 mM HEPES. Before use, KRBH was gassed for 45 min with carbogen (95% O_2_, 5% CO_2_) and then bovine serum albumin (3.5%) and glucose (5.5 mM) were added. Following this, the pH of the buffer was adjusted to 7.4 with NaOH. Minced tissues were incubated at 37°C with gentle agitation (120 orbital strokes/min) for approximately 25–30 min. The digested tissue was then strained using a nylon mesh (500 μm pore size) and cells were transferred to 50 ml plastic tubes, where the stromal vascular fraction (SVF) and mature adipocytes separated into two distinct phases. The SVF sitting at the bottom of the tube was discarded by aspiration and the floating adipocytes were washed 3 times prior to being resuspended in KRBH containing 3.5% fatty acid-free BSA (KRBH-3.5% BSA). In order to distribute an equal number of adipocytes in each treatment condition, cell diameters were measured and total cell numbers determined as previously described [22].

### Measurement of glucose uptake

Glucose uptake was measured either under basal or insulin-stimulated (10 and 100 nM) conditions following the treatment of adipocytes either without (control) or with ALY688. Our initial choice of doses was based on experiments conducted with ALY688 in other cell types (renal fibroblasts, breast cancer cells, etc.) in which concentrations around 100 nM of the compound caused significant effects. Therefore, we decided to start with a similar dose of 100 nM and increase the concentrations to 300 and 500 nM to assess whether higher doses would be required for broader metabolic responses from Epid and Sc Ing adipocytes. We also used AR (10 μM) to test whether another AdipoR agonist would elicit similar effects. Isolated adipocytes (4x10^5^ cells) were transferred to plastic tubes either with or without ALY688 and incubated at 37°C for 1 h. Insulin was added to the medium for the final 20 min of the incubation period. Subsequently, KRB-HEPES containing 0.5 mM 2-deoxy-D-glucose and 0.5 μCi of 2-[1,2-[3]H]deoxy-D-glucose was added to the cells for 3 min, and the incubations were terminated by the addition of cytochalasin B (1.5 mM stock solution). Aliquots of cell suspension (240 μl) were quickly placed in plastic microtubes containing 100 μl of di-‘isononyl’ phthalate. The tubes were centrifuged for 30s (6000 × g) to separate cells from the radioactive incubation medium. Subsequently, fat cells were collected by cutting the tubes through the oil phase and transferring the portion containing the cells to scintillation vials to be counted for radioactivity. Radioactivity of the cells unrelated to glucose transport (non-specific transport) was determined in the same conditions by adding cytochalasin B (50 μM final concentration) to the medium before the addition of 2-[1,2-[3]H]deoxy-D-glucose [23]. Non-Specific values were subtracted from all conditions.

### Measurement of glucose incorporation into lipids

Glucose incorporation into lipids was measured in isolated adipocytes (5 x 10^5^ cells) from the Epid and Sc Ing fat depots as previously described [24]. Briefly, cells were incubated in KRBH-3.5% BSA containing 0.2 μCi/ml of D-[1-^14^C] glucose and 5.5 mM non-labelled D-glucose for 1 h either in the absence or presence of ALY688 (100 and 300 nM) and in the absence or presence of insulin (100 nM). Lipids were then extracted according to the method of Dole and Meinertz [25] and assessed for radioactivity [24].

### Measurement of glucose and palmitate oxidation

Glucose and palmitate oxidation as measures of oxidative capacity were assessed by production of [14]CO_2_ in Epid and Sc Ing isolated adipocytes (5 x 10^5^ cells) as previously described [26]. In order to measure palmitate oxidation, cells were incubated in KRBH-3.5% BSA containing 0.2 μCi/ml of [1–^14^C] palmitic acid and 200 μM non-labelled palmitate. For glucose oxidation, cells were incubated with 0.2 μCi/ml of D-[U-^14^C] glucose and 5.5 mM non-labelled D-glucose. Glucose and palmitate oxidation were measured during 1 h either in the absence or presence of ALY688 (100 nM and 300 nM). For glucose oxidation only, cells were also incubated in the absence or presence of insulin (100 nM). Following 1 h of incubation, the media were acidified with 0.2 ml of H_2_SO_4_ (5 N), and the vials were maintained sealed at 37°C for an additional 1 h for the collection of [14]CO_2_ released from the cells and the media. Each vial used for incubation had a centred isolated well containing a loosely folded piece of filter paper that was moistened with 0.2 ml of 2-phenyl- ethylamine/methanol (1:1, *vol:vol*) to capture [14]CO_2_. At the end of the incubation, the filter paper was removed and transferred to a scintillation vial for radioactivity counting [26].

### Determination of lipolysis

Lipolysis was measured in isolated Epid and Sc Ing adipocytes (6 x 10^5^ cells). Adipocytes were incubated in the absence or presence of ALY688 and with or without 100 nM isoproterenol (β-non-specific agonist) [27]. Each condition was assayed in triplicates and the samples were incubated for 75 min at 37°C with gentle agitation (80 orbital strokes/min). After incubation, a 200 μl aliquot of medium was taken from each vial for the determination of glycerol concentration.

### RNA isolation and quantitative PCR

Primers were designed using the software PrimerQuest (IDT) based on probe sequences available at the Affymetrix database (NetAffx™ Analysis Centre, http://www.affymetrix.com/analysis) for each given gene. RNA was isolated from adipose tissue and isolated adipocytes using the RNeasy Lipid Tissue Kit (Germantown, MD, USA) and complimentary DNA (cDNA) was made from 2 μg of extracted RNA using the ABM EasyScript^TM^ cDNA Synthesis kit (Diamed, Mississauga, ON, Canada), according to the manufacturer’s instructions. Samples were run in duplicate on 96-well plates, and each 20 μl reaction contained 4 μl of cDNA, 0.4 μl of primer, 10 μl of Brightgreen 2x qPCR Mastermix (Diamed, Mississauga, ON, Canada) and 5.6 μl of RNA-free water. Real-time PCR analysis was performed using a Bio-Rad CFX96 Real Time PCR Detection System (Bio-Rad, Mississauga, ON, Canada) using the following amplification conditions: 95°C (10 min); 40 cycles of 95°C (15 s), 60°C (60 s). Values are presented as fold increases relative to GAPDH gene expression [28]. The primers used to probe for rat AdipoR1 and AdipoR2 mRNA levels using qPCR were as follows:

AdipoR1: Forward: 5ʹ-ACTTTGTAACCTGGCGGATGACAG-3ʹ

Reverse: 5ʹ-ATCAAGGCGTGGCTTTGTTTGTCC-3ʹ

AdipoR2: Forward: 5ʹ-AATGATGGCGTTTCTCTCTGGTGC-3ʹ

Reverse: 5ʹ-CACACTTCTTGCGAACGGCATTCA-3ʹ

GAPDH: Forward: 5ʹ-TGACTCTACCCACGGCAAGTTCAA-3ʹ

Reverse: 5ʹ-ACGACATACTCAGCACCAGCATCA-3ʹ

### Western blotting analysis of content and phosphorylation of proteins

*–* Isolated adipocytes from the Epid and Sc Ing fat depots were incubated in the absence or presence of ALY688 for 30 min and then immediately homogenized in a buffer containing 25 mM Tris-HCl, 25 mM NaCl (pH 7.4), 1 mM MgCl_2_, 2.7 mM KCl, 1% Triton X-100 and protease and phosphatase inhibitors (Roche Diagnostics GmbH, Mannheim, Germany). Homogenates were centrifuged, the infranatant collected, and an aliquot was used to measure protein by the Bradford method. Samples were diluted 1:1 (vol:vol) with 2x Laemmli sample buffer and heated to 95°C for 5 min. 25 μg of protein were loaded in each well. Samples were then subjected to SDS-PAGE, transferred to PVDF membrane, and probed for the proteins of interest. All primary antibodies were used at a dilution of 1:1,000. All densitometry analyses were performed using the Scion Image program.

### Statistical analyses

*–* Statistical analyses were performed by either unpaired two-tailed t-tests or one and two-way analysis of variance (ANOVA) with Bonferroni post-hoc tests using the GraphPad Prism statistical software program. Bars represent mean ± SEM. Statistical significance was set at p < 0.05.

## Results

### AMPK and ACC phosphorylation

**Figure 2.**
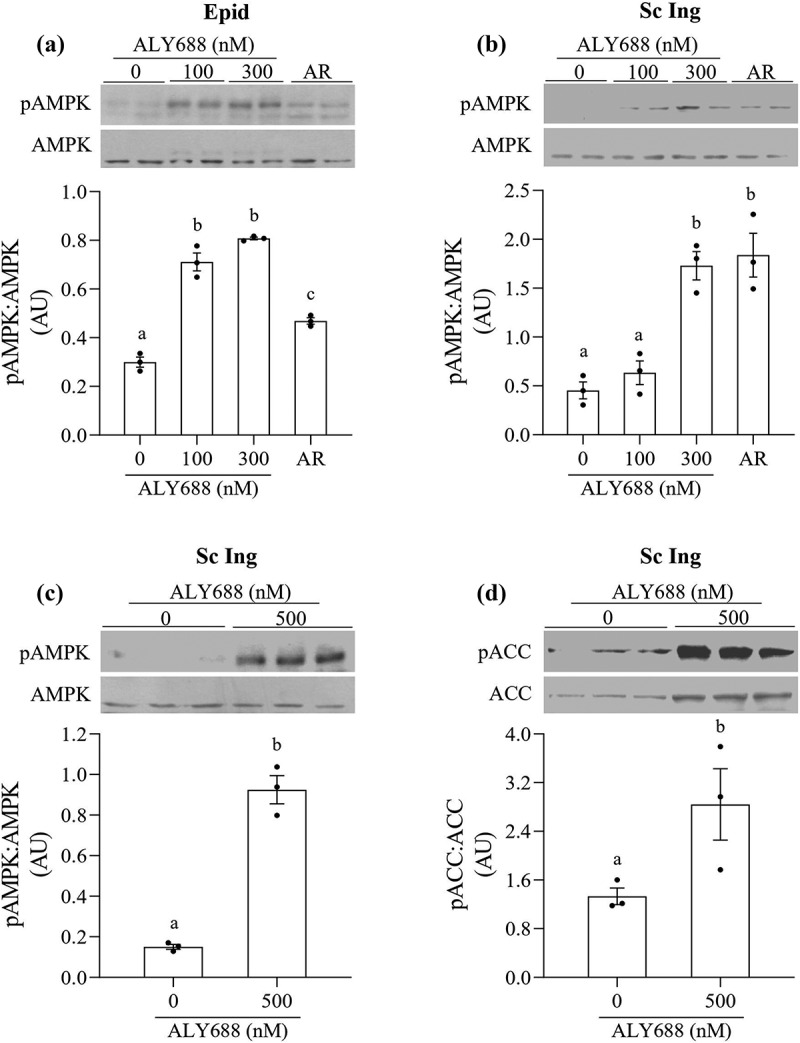
ALY688 (100 and 300 nM) induced AMPK phosphorylation in epididymal (Epid, **A**) and subcutaneous inguinal (Sc Ing, **B**) adipocytes. AdipoRon (AR) was used as a positive control. In Sc Ing adipocytes, a higher dose of ALY688 also increased the phosphorylation of AMPK (c) and ACC (d). Different letters denote statistical significance (p < 0.05). One-way ANOVA for A-B and t-test for C-D, n = 3

– We detected an increase in AMPK phosphorylation when adipocytes were incubated with 100, 300, and 500 nM of ALY688. In Epid adipocytes, 100 and 300 nM of ALY688 significantly increased AMPK phosphorylation by 2.37 and 2.7-fold, relative to 0 nM, respectively, whereas AR (10 µM) increased this variable by 1.57-fold (Figure 2(a)). In Sc Ing adipocytes, 300 nM of ALY688 increased AMPK phosphorylation by 3.81-fold relative to 0 nM, which was of similar magnitude to the AR effect in these cells (Figure 2(b)). However, an increase in AMPK phosphorylation was not observed in this depot with 100 nM of the drug (Figure 2(b)). To confirm that phosphorylation of AMPK was accompanied by an increase in its activity, we also assessed the phosphorylation of ACC, a downstream target of AMPK. In the presence of 500 nM of ALY688, AMPK phosphorylation increased 6.2-fold (Figure 2(c)), and this was accompanied by a 2.13-fold increase in ACC phosphorylation (Figure 2(d)). The generation of cell lysates from primary adipocytes requires a large amount of adipose tissue; thus, in order to optimize the preparation, we used the Sc Ing fat depot where the amount of tissue available and the yield of cells were the highest. Also, in order to have three replicates for control and the compound, we used only one dose of ALY688 (500 nM) as shown in Figure 2(c and d).

### Glucose uptake

**Figure 3.**
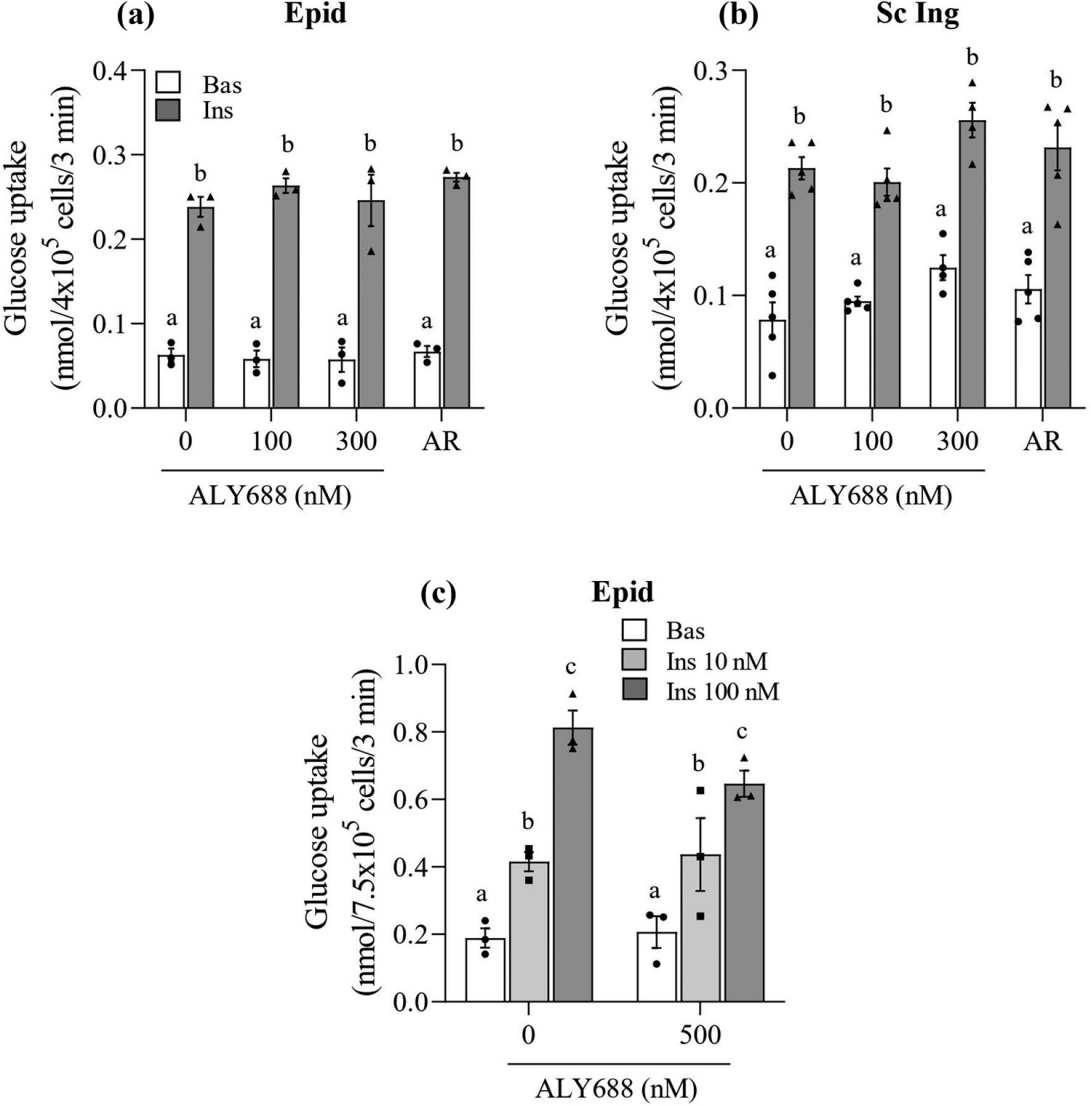
No effect of ALY688 (100, 300, and 500 nM) on basal (Bas) and insulin (Ins, 100 nM)-stimulated glucose uptake in epididymal (Epid, **A**) and subcutaneous inguinal (Sc Ing, **B**) adipocytes. Glucose uptake was also assessed in Epid adipocytes exposed to 500 nM of ALY688 for 60 min (c) and then stimulated with 10 and 100 nM of insulin. Different letters denote statistical significance (p < 0.05). Two-way ANOVA, n = 3–5

As expected, glucose uptake was increased upon insulin stimulation in both Sc Ing and Epid adipocytes. However, no significant effects of any concentration of either ALY688 or AR (10 µM) on basal and insulin-stimulated glucose uptake were detected (Figure 3(a and b)). Because supraphysiological insulin concentrations (100 nM) were used in the initial experiments, it could be that glucose uptake was maximized, masking a potential effect of ALY688 on this variable. To explore this, we conducted additional experiments using a lower (more within the physiological range, 10 nM) insulin concentration. Also, we used a higher concentration of the compound (500 nM) to test whether this would be more effective in adipocytes than the previous doses used (Figure 3(c)). Despite this, we found that even under lower doses of insulin, glucose uptake was not significantly affected by 500 nM of ALY688 in Epid adipocytes (Figure 3(c)), and similar results were also found for Sc Ing adipocytes.

### AKT and p38 MAPK phosphorylation

**Figure 4.**
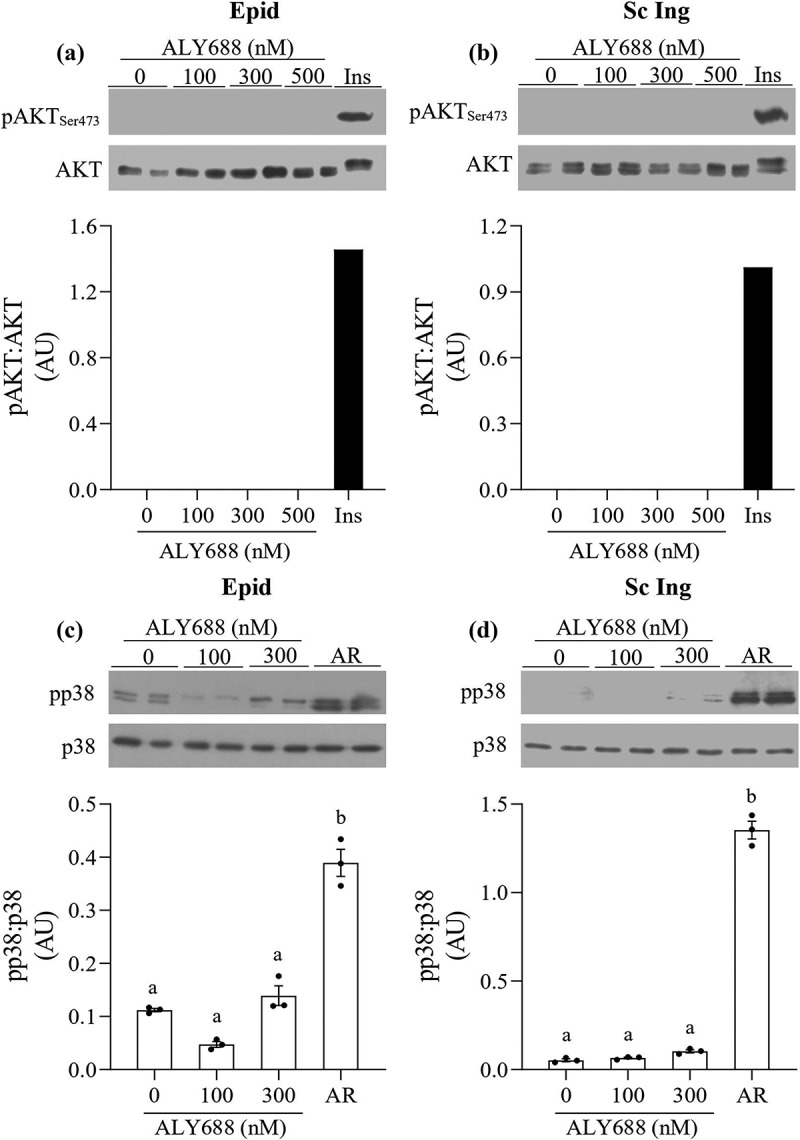
ALY688 did not affect AKT_Ser473_ and p38 MAPK phosphorylation in Epid (a and c) and Sc Ing adipocytes (b and d), whereas AdipoRon (AR) and insulin induced robust increases in AKT_Ser473_ and p38 MAPK phosphorylations in adipocytes from both fat depots. Different letters denote statistical significance (p < 0.05). One-way ANOVA, n = 3

To test whether major steps of the insulin signalling pathway were affected by ALY688, we measured AKT_Ser473_ phosphorylation in both Epid and Sc Ing adipocytes exposed to 0, 100, 300, and 500 nM of the compound. No effect of ALY688 was detected on AKT_Ser473_ phosphorylation in Epid (Figures 4(a) and Sc Ing (Figure 4(b)) adipocytes, which is consistent with the lack of effect on glucose uptake in these cells. As a positive control, cells isolated from both fat depots were also treated with insulin (100 nM), and it caused a robust AKT_Ser473_ phosphorylation response to the hormone (Figure 4(a and b)). Additionally, because adiponectin initiates a series of tissue-dependent signal transduction events through distinct signalling pathways, including p38 MAPK [7], we also tested whether this would be the case in Epid and Sc Ing adipocytes. As shown in Figure 4(c and d), p38 phosphorylation was not affected by 100 and 300 nM of ALY688 in Epid and Sc Ing adipocytes. However, a robust induction of p38 MAPK phosphorylation was detected when adipocytes from both fat depots were incubated with 10 µM of AR (Figure 4(c and d)).

### Glucose incorporation into lipids and glucose oxidation

**Figure 5.**
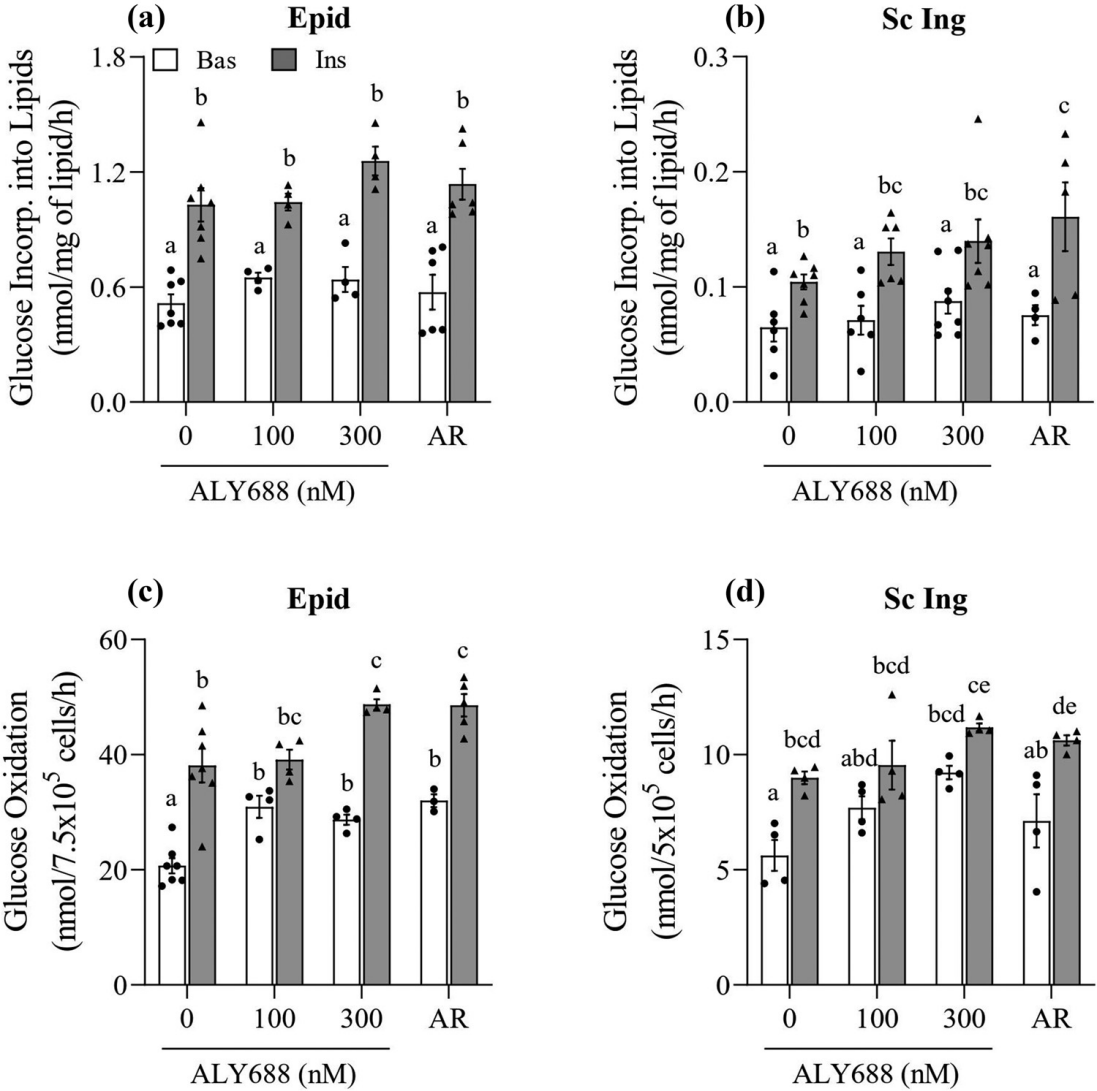
ALY688 (100 and 300 nM) did not affect glucose incorporation into lipids (a and b), but it enhanced in a dose-dependent and tissue-specific manner basal (Bas) and insulin (Ins)-stimulated rates of glucose oxidation in Epid (c) and Sc Ing (d) adipocytes. Different letters denote statistical significance (p < 0.05). Two-way ANOVA, n = 3–7

ALY688 did not affect glucose uptake in Epid and Sc Ing adipocytes; however, it could still affect metabolism of this substrate inside the cells. To evaluate this, we measured the incorporation of glucose into lipids and also glucose oxidation in Epid and Sc Ing adipocytes under basal and insulin-stimulated conditions. We found that neither Epid nor Sc Ing rates of glucose incorporation into lipids (lipogenesis) were affected by the compound (Figure 5(a and b)). However, ALY688 caused dose and tissue-specific effects on glucose oxidation in Epid and Sc Ing adipocytes. This was characterized by significantly enhanced basal glucose oxidation by 49% and 39% in Epid adipocytes in the presence of 100 and 300 nM of ALY688, respectively, when compared to cells incubated in the absence of insulin and ALY688 (Figure 5(c)). In Sc Ing adipocytes, 100 and 300 nM of ALY688 increased basal glucose oxidation by 37% and 64%, respectively, with only the latter concentration reaching statistical significance when compared to 0 nM ALY688 (Figure 5(d)).Insulin-stimulated glucose oxidation was also enhanced by the compound, although only in Epid adipocytes treated with 300 nM of ALY688 or AR (Figure 5(c)).

### Palmitate oxidation

**Figure 6.**
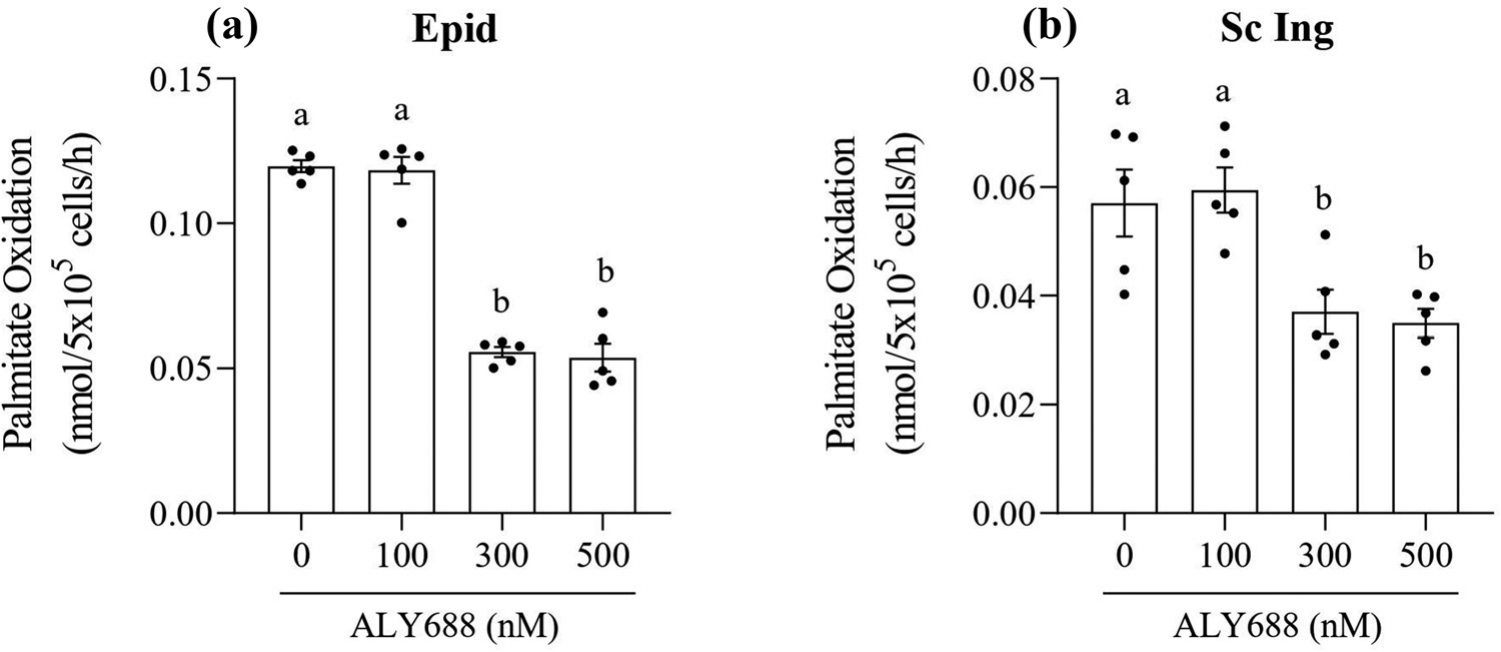
As the concentration of ALY688 increased beyond 100 nM, rates of palmitate oxidation were reduced in both epididymal (Epid, **A**) and subcutaneous inguinal (Sc Ing, **B**) adipocytes. Different letters denote statistical significance (p < 0.05). One-way ANOVA, n = 5

Because ALY688 induced a consistent increase in AMPK and ACC phosphorylation in isolated adipocytes from both fat depots, we tested whether such effect was also accompanied by a functional metabolic alteration in these cells. The initial expectation was that increased AMPK phosphorylation would increase rates of fatty acid oxidation, as it is frequently reported in skeletal muscle cells [8,14]. However, to our surprise, 300 and 500 nM of ALY688 suppressed basal palmitate oxidation by ~55% and ~35% in both the Epid and Sc Ing adipocytes, respectively, relative to the 0 nM group (Figure 6(a and 6b)). No effect on palmitate oxidation was observed in either depot with 100 nM of ALY688 (Figure 6(a and b)).

### Lipolysis

**Figure 7.**
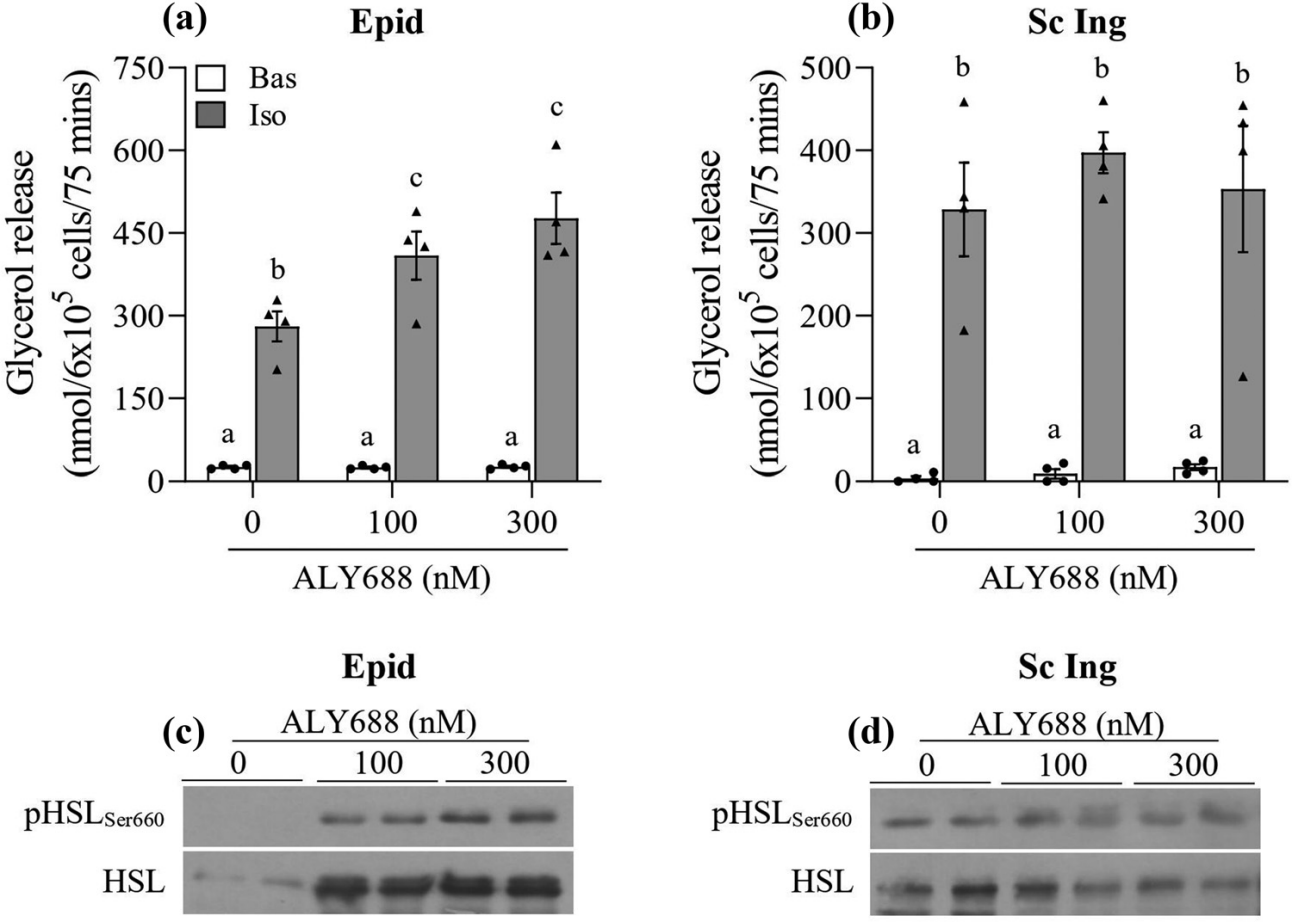
Lipolysis was affected in tissue-specific manner by ALY688. In Epididymal (Epid) adipocytes, isoproterenol (Iso)-stimulated lipolysis increased (a), whereas no effect of ALY688 (100 and 300 nM) was detected in Sc Ing (b) adipocytes either under basal (Bas) or Iso-stimulated conditions. Similarly, HSL phosphorylation was elevated in Epid (c), but not in Sc Ing (d) adipocytes. Different letters denote statistical significance (p < 0.05). Two-way ANOVA, n = 4 for glycerol release and n = 2 for HSL content and phosphorylation

In order to assess whether ALY688 could affect the ability of adipocytes to export fatty acids, we also measured lipolysis in these cells. We found that basal lipolysis was not affected by treating cells with ALY688; however, when Epid adipocytes were stimulated with 100 and 300 nM of ALY688 and 100 nM of isoproterenol, glycerol release increased by 45% and 70%, respectively, when compared to cells incubated with isoproterenol only (Figure 7(a)). In Sc Ing adipocytes, ALY688 had no significant effect on rates of glycerol release either under basal or isoproterenol (Iso)-stimulated conditions (Figure 7(b)). In order to assess potential mechanisms by which ALY688 enhanced Iso-stimulated glycerol release in Epid adipocytes, we measured phosphorylation of hormone sensitive lipase (HSL_Ser660_) in both Epid and Sc Ing adipocytes. As depicted in Figure 7(c and d), ALY688 increased phosphorylation of HSL_Ser660_ in Epid, but not in Sc Ing adipocytes. This suggests that ALY688-induced HSL phosphorylation could be linked to the increase in glycerol release seen in Epid adipocytes. However, we could not perform a statistical analysis because we had only 2 samples probed for HSL phosphorylation. Furthermore, even though HSL plays a very important role in adipose tissue lipolysis, adipose triglyceride lipase (ATGL) activity could also be affected by ALY688. However, the activity of this latter lipase was not measured in this study.

### Adiponectin receptor gene expression in isolated adipocytes and whole WAT

**Figure 8.**
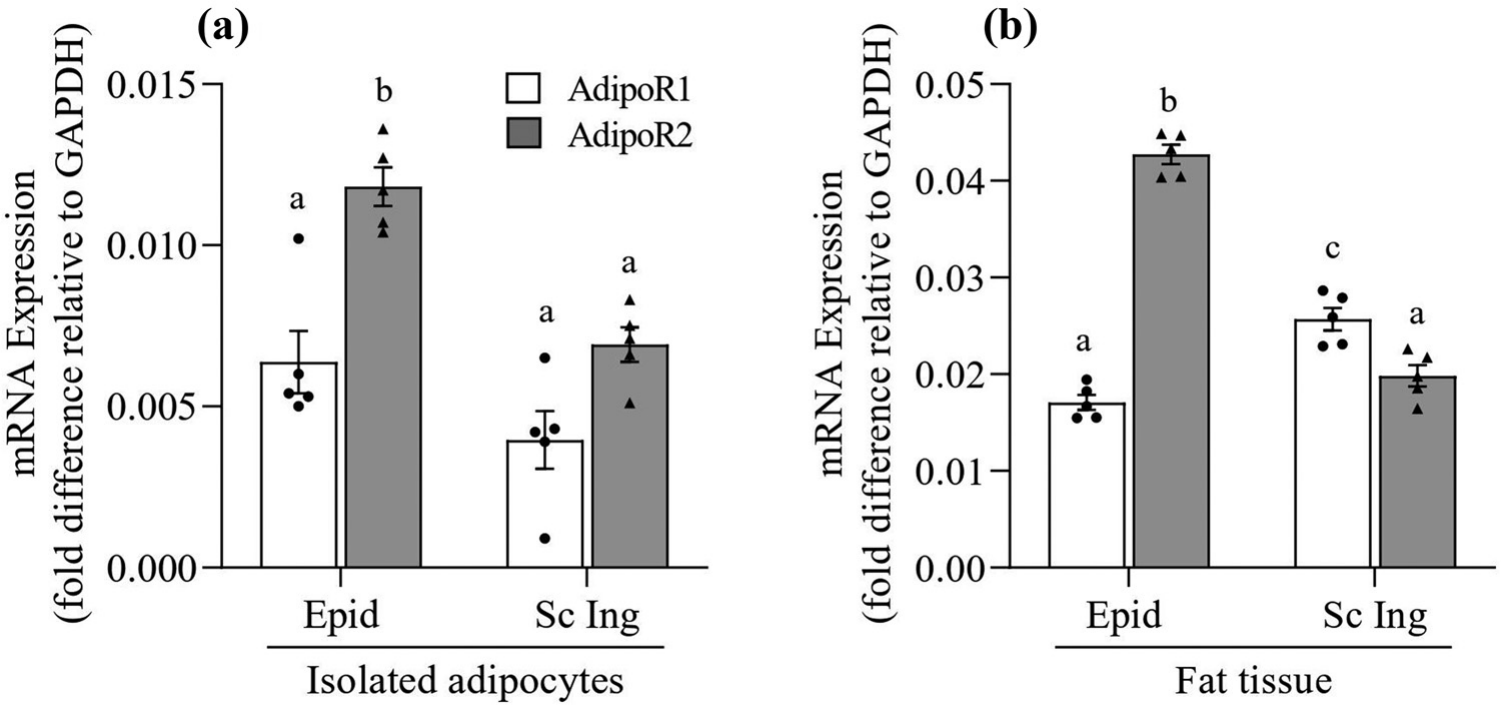
Distinct profile of AdipoR1 and AdipoR2 mRNA expression between epididymal (Epid) and subcutaneous inguinal (Sc Ing) adipocytes (a) and fat depots (b). Different letters denote statistical significance (p < 0.05). Two-way ANOVA, n = 5

In isolated adipocytes, we found that the mRNA expression of AdipoR2 was 1.85-fold and 1.75-fold higher than AdipoR1 in Epid and Sc Ing cells, respectively (Figure 8(a)). Furthermore, the mRNA expression of both receptors was significantly lower in Sc Ing than Epid cells. In fact, AdipoR1 and AdipoR2 mRNA expressions were ~45% lower in Sc Ing than in Epid adipocytes (Figure 8(a)). In whole Epid fat tissue, AdipoR2 mRNA expression was 2.5-fold higher than AdipoR1, which was similar to what we found in isolated cells. However, in the Sc Ing fat pad the reverse was found, with AdipoR1 mRNA expression being 1.3-fold higher than AdipoR2 (Figure 8(b)). All our experiments were conducted in isolated cells; therefore, it is possible that the higher expression of AdipoR2 in Epid adipocytes dictated the distinct metabolic responses that ALY688 treatment elicited in isolated cells from visceral and subcutaneous fat depots.

## Discussion

Our findings provide evidence that acute exposure of isolated Epid and Sc Ing rat adipocytes to ALY688 did not alter AKT and p38 MAPK phosphorylation, basal and insulin-stimulated glucose uptake, and glucose incorporation into lipids (lipogenesis) in these cells. However, ALY688 treatment increased AMPK and ACC phosphorylation, although this was accompanied by suppression instead of stimulation of palmitate oxidation in Epid and Sc Ing adipocytes. This latter finding is contrary to what has been demonstrated in skeletal muscle cells under adiponectin-stimulated AMPK activation [8,14], suggesting a distinct regulatory role for adiponectin-mediated AMPK activation in WAT glucose and fat metabolism in comparison to liver and skeletal muscle. In fact, ALY688 enhanced rates of glucose oxidation in a concentration-dependent and tissue-specific manner in Epid and Sc Ing adipocytes. Basal rates of glucose oxidation consistently increased in cells from both fat depots, whereas under insulin-stimulated conditions only higher concentrations (300 nM) of the compound elicited an effect in Epid adipocytes. The data indicate that acute ALY688 treatment diverts glucose towards oxidation in adipocytes, which could positively contribute to whole-body glycaemic control. We have also found that both AR and ALY688 enhanced AMPK phosphorylation in Epid and Sc Ing adipocytes, whereas only AR increased p38 MAPK phosphorylation in these cells. However, because neither ALY688 nor AR affected either basal or insulin-stimulated uptake of glucose and its incorporation into lipids, the ability of AR to induce p38 MAPK phosphorylation did not seem to have any direct impact on glucose metabolism in adipocytes from both fat depots.

There are at least three potential explanations for the reduction in fatty acid oxidation despite ALY688 inducing AMPK phosphorylation in isolated adipocytes: 1) it could be that enhanced glucose oxidation provided energy for the cells and caused a shift in fatty acid metabolism away from oxidation, even though AMPK phosphorylation was increased by ALY688. This could be possible because there is a relatively small number of mitochondria in white adipocytes, which normally limits the ability of these cells to elicit an elevated rate of fat oxidation [21,29]. However, because ALY688 consistently increased basal glucose oxidation in Epid and Sc Ing adipocytes, this must have led to a condition in which even fewer fatty acids were required and used for β-oxidation and energy production in these cells. Therefore, a shift in substrate utilization seems a plausible mechanism by which ALY688 led to the suppression of palmitate oxidation, despite increased AMPK phosphorylation in adipocytes; 2) enhancement of fatty acid oxidation by adiponectin in skeletal muscle cells has been shown to depend on the sequential activation of AMPK and p38 MAPK, leading to induction of PPARα transcriptional activity and of its target oxidative genes (*e.g*. ACO and CPT1) [7]. In our studies, even though ALY688 caused AMPK activation in Epid and Sc Ing adipocytes, p38 MAPK phosphorylation was not affected by the compound. Thus, it could be that ALY688 promoted only partial activation of signalling events required for the enhancement of fatty acid oxidation in adipocytes. Under these conditions, glucose was clearly the preferred substrate for oxidation in these cells, which, as previously described, likely led to fewer fatty acids being diverted for oxidation; and finally, 3) it is also possible that fatty acid uptake was impaired under concentrations of ALY688 above 100 nM. This could be an AMPK-independent dose-dependent effect of the compound that suppressed specific adipocyte functions.

ALY688 did not affect basal rates of lipolysis either in Epid or Sc Ing adipocytes; however, isoproterenol-stimulated lipolysis increased in Epid adipocytes treated with ALY688. The mechanism underlying this depot-specific enhancement of beta-adrenergic-stimulated lipolysis by ALY688 seems to involve an increase in HSL phosphorylation. These findings differ from previous reports in which adiponectin suppressed lipolysis via inhibition of HSL phosphorylation in mouse adipocytes [12], and that a collagen domain-derived short adiponectin peptide promoted adipogenesis in 3T3L1 adipocytes [11]. These discrepancies could be attributed to methodological differences between studies. For instance, a lower lipolytic response was previously reported in Epid adipocytes from Adipoq^−/-^ mice exposed to BRL37344 (a selective β3-adrenergic agonist), as well as in 3T3L1 adipocytes maintained in co-culture with FAO cells overexpressing full-length mouse adiponectin [12]. In this study, we performed acute (1 h) incubations of primary rat adipocytes in the presence of a peptide-based adiponectin mimetic and then stimulated the cells with isoproterenol, a non-specific β-adrenergic agent.

From our metabolic studies, it was clear that ALY688 caused tissue-specific and dose-dependent effects on glucose and fat metabolism in adipocytes. In general, Epid adipocytes elicited more pronounced responses to ALY688 than Sc Ing cells, particularly with respect to enhancing glucose oxidation and lipolysis while suppressing palmitate oxidation. Under these conditions, ALY688 could confer metabolic flexibility to the WAT and control WAT expansion, an effect that has been previously attributed to adiponectin [3,13]. This would allow for depot-specific exportation of fatty acids for utilization in peripheral tissues under adrenergic-stimulated conditions, while also enhancing glucose oxidation within the WAT. It could be that the tissue-specific responses resulted from distinct expressions of adiponectin receptors (AdipoR1 and AdipoR2) between Epid and Sc Ing cells. Therefore, we determined the expression profile of AdpoR1 and AdipoR2 in Epid and Sc Ing adipocytes, as well as in intact adipose tissue. It has been reported that it is AdipoR2 that carries out the effects of adiponectin in WAT [4]. Indeed, we found that in isolated Epid and Sc Ing adipocytes, AdipoR2 mRNA expression was approximately two-fold higher than AdipoR1. Also, Epid adipocytes displayed significantly higher AdipoR2 mRNA expression than Sc Ing adipocytes. Similarly, when analysing whole fat tissue, we also detected much higher AdipoR2 mRNA expression in the Epid than in the Sc Ing fat depot. However, in the Sc Ing fat depot AdipoR1 mRNA expression was slightly higher than AdipoR2 mRNA expression, which could be attributed to the presence of a higher population of non-adipocyte cells in the Sc Ing than in the Epid fat depot. Because our experiments were conducted in isolated adipocytes, it is plausible that the more pronounced responses of Epid than Sc Inc adipocytes to ALY688 resulted from a much lower expression of AdipoR2 in the latter than the former adipocytes.

In summary, we found that acute treatment with ALY688 increased AMPK phosphorylation in Epid and Sc Ing rat adipocytes; however, this was accompanied by suppression of fat oxidation and no alteration in basal and insulin-stimulated glucose uptake in these cells. Furthermore, ALY688 increased rates of glucose oxidation in Epid and Sc Ing adipocytes and enhanced adrenergic-stimulated lipolysis, although the latter effect was only detected in Epid adipocytes. These findings provide evidence that the AdipoR agonist ALY688 can regulate adiposity and affect glycaemic control by altering substrate portioning in adipocytes in a fat depot-specific manner.
